# Body Composition-Specific Asthma Phenotypes: Clinical Implications

**DOI:** 10.3390/nu14122525

**Published:** 2022-06-17

**Authors:** Xin Zhang, Ke Deng, Yulai Yuan, Lei Liu, Shuwen Zhang, Changyong Wang, Gang Wang, Hongping Zhang, Lei Wang, Gaiping Cheng, Lisa G. Wood, Gang Wang

**Affiliations:** 1 Pneumology Group, Department of Integrated Traditional Chinese and Western Medicine, Clinical Research Center for Respiratory Disease, West China Hospital, Sichuan University, Chengdu 610044, China; zhangxinwch@126.com (X.Z.); liuleiwch@126.com (L.L.); zsw1609925175@163.com (S.Z.); wanggang-cn@foxmail.com (G.W.); zhongping921@126.com (H.Z.); wanglei@medmail.com.cn (L.W.); 2 Department of Respiratory and Critical Care Medicine, Clinical Research Center for Respiratory Disease, West China Hospital, Sichuan University, Chengdu 610044, China; dengke_18@126.com (K.D.); wangchangyong0109@163.com (C.W.); 3 Laboratory of Pulmonary Immunology and Inflammation, Frontiers Science Center for Disease-Related Molecular Network, Sichuan University, Chengdu 610213, China; 4 Department of Respiratory Medicine, Traditional Chinese Medicine Hospital Affiliated to Southwest Medical University, Luzhou 646699, China; yuanyl@swmu.edu.cn; 5 Institute of Environmental Medicine, Karolinska Institute, 11883 Stockholm, Sweden; 6 Department of Clinical Science and Education, Södersjukhuset, Karolinska Institute, 11883 Stockholm, Sweden; 7 Department of Clinical Nutrition, West China Hospital, Sichuan University, Chengdu 610044, China; cgphello@163.com; 8 Priority Research Center for Healthy Lungs, Hunter Medical Research Institute, University of Newcastle, New Lambton, NSW 2308, Australia; lisa.wood@newcastle.edu.au

**Keywords:** asthma, phenotype, body composition, nutritional status, skeletal muscle mass

## Abstract

Background: Previous studies have indicated the limitations of body mass index for defining disease phenotypes. The description of asthma phenotypes based on body composition (BC) has not been largely reported. Objective: To identify and characterize phenotypes based on BC parameters in patients with asthma. Methods: A study with two prospective observational cohorts analyzing adult patients with stable asthma (*n* = 541 for training and *n* = 179 for validation) was conducted. A body composition analysis was performed for the included patients. A cluster analysis was conducted by applying a 2-step process with stepwise discriminant analysis. Logistic regression models were used to evaluate the association between identified phenotypes and asthma exacerbations (AEs). The same algorithm for cluster analysis in the independent validation set was used to perform an external validation. Results: Three clusters had significantly different characteristics associated with asthma outcomes. An external validation identified the similarity of the participants in training and the validation set. In the training set, cluster Training (T) 1 (29.4%) was “patients with undernutrition”, cluster T2 (18.9%) was “intermediate level of nutrition with psychological dysfunction”, and cluster T3 (51.8%) was “patients with good nutrition”. Cluster T3 had a decreased risk of moderate-to-severe and severe AEs in the following year compared with the other two clusters. The most important BC-specific factors contributing to being accurately assigned to one of these three clusters were skeletal muscle mass and visceral fat area. Conclusion: We defined three distinct clusters of asthma patients, which had distinct clinical features and asthma outcomes. Our data reinforced the importance of evaluating BC to determining nutritional status in clinical practice.

## 1. Introduction

In recent years, the importance of body composition in the development and progression of asthma has become increasingly recognized. In particular, obesity has gained focus, as a common asthma-related comorbidity, which increases the prevalence and incidence of asthma [1,2,3]. Obese patients tend to have more severe asthma than lean patients, with a 4- to 6-fold higher risk of being hospitalized compared with lean patients with asthma [4]. In the United States, nearly 60% of adults with severe asthma are obese [5]. The progression of asthma and obesity are closely linked [6,7]. There is evidence that obese asthmatics have worse asthma control, lower quality of life [8], and do not respond as well to standard controller medications for asthma [9]. The comorbidity of obesity in certain patients with asthma has recently been identified as a unique asthma phenotype “obese asthma” [10].

One of the main limitations in studying the role of obesity and body composition emerges from the use of body mass index (BMI, weight relative to height, expressed as kg/m^2^). As a simple measurement, BMI is widely used to categorize nutritional status. Although there is an association between BMI and fat mass or percentage of body fat (PBF), BMI cannot be considered as a good proxy of fat mass [11]. Studies have indicated the possible limitations of using BMI, which cannot distinguish between muscle and fat tissue [12]. Additionally, the sensitivity and specificity of BMI for detecting people with excess PBF are poor [13,14].

To overcome these limitations within the use of BMI, body composition analysis (BCA) has been used to further explore metabolic and nutritional status. The wide use of BCA has enabled the improvement of nutritional evaluation and increased the recognition of impaired nutritional status by clinicians. BC evaluation has been studied in various populations and diseases [2,12]. Our previous studies found that compared to BCA, BMI was of limited importance for assessing asthma [2]. Recent studies have highlighted the relationship between BC parameters (fat mass (FM), PBF, and skeletal muscle mass (SMM)) with poorer nutritional status [15]. By allowing for the early detection of undernutrition, BC evaluation has been shown to improve the clinical outcomes for some diseases [15].

In recent years, there has been an increasing interest in the heterogeneity and phenotyping of asthma using cluster analysis in different asthma populations [16,17,18,19,20]. However, despite these well-conducted cluster analyses, the identification of phenotypes based on measurements of BC and nutritional status has not been previously reported. By using BCA, we can define asthmatic phenotypes characterized by differences in the nutritional status of BC parameters: FM, PBF, visceral fat area (VFA), and SMM. Moreover, evaluating nutritional status by BCA and further exploring asthma phenotypes can help uncover the significance of nutritional status in the assessment, management, and progression of asthma. Thus, this study aimed to identify and characterize phenotypes based on anthropometric, clinical, and BC parameters in people with asthma. We hypothesized that BC parameters can guide the classification of clinical asthma phenotypes and provide valuable information to improve asthma management.

## 2. Materials and Methods

### 2.1. Study Design and Participants

**Figure 1:**
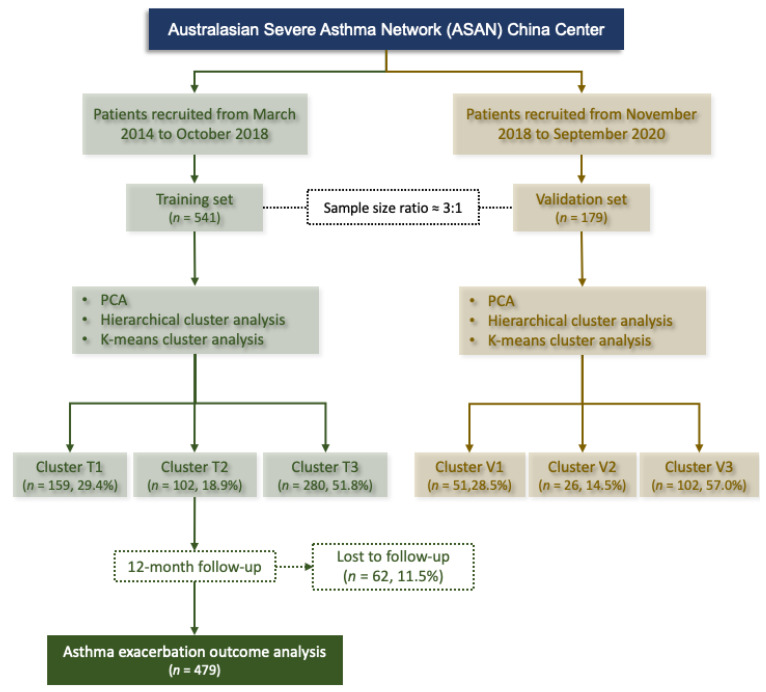
Flowchart for patient inclusion in the training and validation set. Cluster T, clusters identified in the training set; Cluster V, clusters identified in the validation set; PCA, principal component analysis.

The ASAN (https://www.severeasthma.org.au, accessed on 13 June 2022) is a multicenter clinical research network (Australia, Singapore, China, and New Zealand) in a real-world setting. This study included two prospective observational cohort studies which were conducted from March 2014 to October 2018 (541 patients; cohort 1 as training set) and November 2018 to September 2020 (179 patients; cohort 2 as validation set) (Figure 1). The sample size ratio of training set and validation set was 3:1. Adults (≥18 years old) with a diagnosis of stable asthma according to the Global Initiative for Asthma (GINA) (21) criteria were consecutively recruited at the clinic of West China hospital, China (ASAN China Center, Chengdu, China). Stable asthma was defined as no respiratory tract infection and no exacerbation or systemic corticosteroid use in the previous 4 weeks. The inability to understand the questionnaires, perform spirometry or sputum induction, pregnancy, and breastfeeding were also listed as exclusions. Cohort 1 was used for performing cluster analysis (training set) and cohort 2 for validating the clusters identified in training set. Data from training set were used to identify clinical asthma phenotypes by cluster analysis. The patients in training set were followed-up for 12 months to monitor asthma exacerbations (AEs) and to validate the effect of the identified clusters on AEs. To assess whether the cluster analysis in training set had reproducibility, the same algorithm of cluster analysis in training set was used for the validation set. As a real-world study, indications for patient treatment were based on the GINA recommendations [21]. Step-up or step-down treatments were adjusted in a continuous cycle of assessment, treatment, and review. This study was approved by the Institutional Review Board (IRB) at West China Hospital, Sichuan University (Chengdu, China) (No. 2014–30) and registered at Chinese Clinical Trial Registry (ChiCTR-OOC-16009529; https://www.chictr.org.cn, accessed on 13 June 2022). All participants provided written informed consent.

### 2.2. Multidimensional Assessment and Data Collection

Data on demographics and clinical characteristics were collected using standardized case report form. Detailed further assessments including anthropometrics and body composition, spirometry and fractional exhaled nitric oxide, atopy and skin prick tests, sputum induction and peripheral blood collection and detection, asthma exacerbation, and psychological dysfunction (anxiety and depression), were defined and shown in the Supplementary Materials.

#### Measurements of Body Composition

The body composition, including the VFA (cm^2^), FM (kg), PBF (%), and SMM (kg), was measured by a multifrequency bioimpedance analysis (BIA) with the InBody S10 analyzer (Body Composition Analyzer; Biospace Co., Ltd., Seoul, Korea). InBody S10 provides 6 different frequency impedance measurements (1, 5, 50, 250, 500, and 1000 kHz) and 3 different frequencies of phase angle measurement (5, 50, and 250 kHz) at each 5 segments (right arm, left arm, trunk, right leg, and left leg). The BIA measurements were performed by a nutritionist in our research group (Gaiping Cheng) that was trained according to the InBody S10 user’s manual and the recommendations for clinical application of bioelectrical impedance analysis [22]. After height and weight were measured, four electrodes were attached to both upper and lower extremities in the supine position. The patients had an overnight fasting, emptied the bladder by urinating, wore light indoor clothing, and assumed a standing posture during the measurement, during which the ambient temperature remained at 25 °C. Standard ranges for FM, PBF, VFA, and SMM were based on Asian standards in InBody S10 user’s manual. Although dual-energy X-ray (DXA) is considered the gold standard for body composition measurement, BIA and DXA have been reported as strongly correlated [23,24,25].

### 2.3. Statistical Analysis

A total of 366 variables including measurements of body composition were collected and recorded. Variables with missing data (5–40%) were imputed using multiple imputation method (MI). Data below 5% are negligible and more than 40% missing data did not use MI [20,26]. Variable selection process was performed as mentioned in our study [26] and previous studies [27,28,29,30,31] with detailed information in Supplementary Materials. Finally, 10 variables were selected for principal component analysis (PCA) based on the pattern of loading, correlation coefficient, and clinical perspective, including sex (female = 1), age (years), pre-FEV_1_ (%), Hospital Anxiety and Depression scale-anxiety (HADS)-A (scores), HADS-D (scores), BMI (kg/m^2^), FM (kg), PBF (%), VFA (cm^2^), and SMM (kg).

#### 2.3.1. Principal Component Analysis (PCA)

Reducing the dimensionality of the data prior to clustering algorithms reduces the risk of overfitting. Thus, a principal component analysis (PCA) with varimax rotation was performed to merge the variables of interest into a multivariate component. The selection process regarding the appropriate number of PCs [32,33,34,35], the variables restructured for PCs in the training and validation sets, and the findings of PCA [17,36] were shown in Supplementary Materials.

#### 2.3.2. Cluster Analysis

Cluster analysis was conducted by applying a 2-step process using the four PCs identified in the PCA as described in our studies and previous published studies [20,26,37,38,39,40,41,42]. Detailed information about cluster analysis were shown in Supplementary Materials.

#### 2.3.3. Other Analyses

Other statistical analyses were shown in the Supplementary Materials, including the differences of demographic and clinical data between clusters, Pearson or Spearman’s coefficients for assessing correlations, and multiple logistic regression modeling between the uncontrolled asthma and AEs in the following year. Statistical analyses were carried out using SPSS version 23.0 (IBM, Armonk, NY, USA). *p*-value less than 0.05 was considered statistically significant. *p*-values may be adjusted for multiple comparisons of the clusters.

## 3. Results

### 3.1. Training Cohort and Characteristics

A total of 541 patients with asthma were included in the training set and 479 patients (88.5%) completed the one-year follow-up. The characteristics of the training cohort are presented in Table 1. A total of 350 patients (64.7%) were females, with a median age of 49.0 (IQR: 39.0, 58.0) years, and a median BMI of 22.73 (IQR: 20.69, 24.77) kg/m^2^. The prevalence of a family history of asthma and atopy were 35.3% and 44.2%, respectively. The median HADS-D and HADS-A were 1.0 (IQR: 0, 3) and 1.0 (IQR: 0, 4), respectively. There were 248 patients (45.8%) with uncontrolled asthma and 158 patients (29.2%) had experienced at least one severe exacerbation in the past 12 months. The frequency of comorbidities ranged from 1.1% to 56.7%. Rhinitis (56.7%) and eczema (16.8%) were the most prevalent comorbidities. Commonly, undernutrition is characterized by a reduction of the fat-free mass (FFM, mainly SMM) and fat mass (FM) [12,15]. The mean FM, SMM, PBF, and VFA were 16.83 (SD: 6.13) kg, 22.69 (SD: 4.73) kg and 28.36 (SD:7.41) %, and 75.64 (SD:31.73) cm^2^, respectively (Table 1).

### 3.2. Cluster Analysis and Description

According to the PCA, the Kaiser–Meyer–Olkin (0.648) and the Bartlett’s Test of Sphericity (*p* < 0.001) confirmed that the cluster analysis was appropriate. The PCA identified four components: component 1 encompassed the variables of BMI, FM, PBF, and VFA; component 2 encompassed sex and SMM; component 3 encompassed HADS-D and HADS-A; and component 4 encompassed age and pre-FEV1% (Table S1). Cluster analysis: Ward’s cluster analysis was based on the significant components identified by the PCA. Using the hierarchical cluster analysis described in the Methods, based on the pseudo-F statistic and Pseudo-T2 statistic (Table S2), three clusters were identified. A silhouette plot indicated a reasonable structure of our cluster analysis (Silhouette Coefficient (SC) = 0.58) [37] (Figure S1).

#### 3.2.1. Cluster T1 (Cluster 1 in the Training Set): Patients with Undernutrition

Cluster Training (T) 1 (*n* = 159, 29.4%) contained older patients (59.0 (51.0, 68.0, *p* < 0.05) years and a greater history of family asthma (*n* = 72, 45.3%, *p* < 0.017) compared with cluster T2 and T3, but less eosinophilic asthma (*n* = 86, 54.1%, *p* < 0.005) (Table 1) compared with T3.

**Figure 2:**
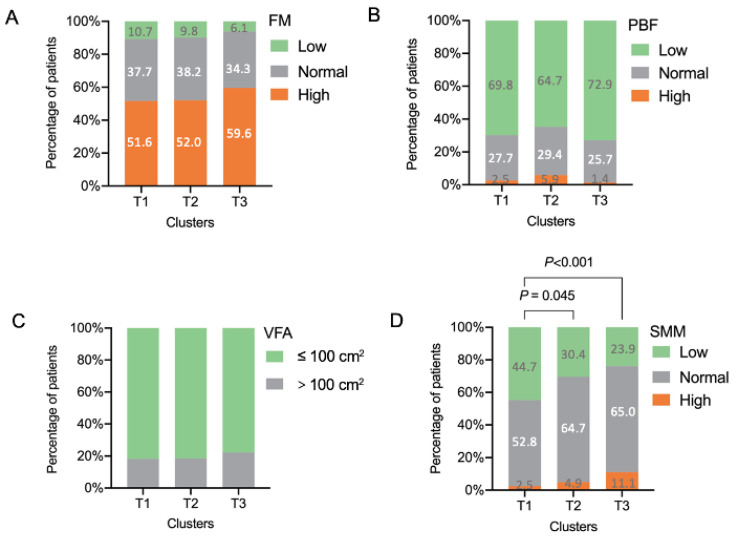
Body composition of the included patients with asthma grouped by cluster analysis. (**A**) FM; (**B**) PBF; (**C**) VFA; (**D**) SMM. FM, fat mass; PBF, percentage body fat; SMM, skeletal muscle mass; VFA, visceral fat area. Standard ranges for FM, PBF, VFA, and SMM were based on Asian standards in InBody S10 user’s manual.

In our study, patients in cluster T1 presented with lower BMI (22.36 (19.97, 24.15) kg/m^2^), FM (15.24 (5.41) kg), PBF (27.24 (7.50) %), VFA (70.66 (29.84) cm^2^) (*p* < 0.05) (Table 1), and lower SMM (21.57 (4.07) kg) and proportion of patients in the low level of SMM (44.7%, *p* < 0.001) (Figure 2) compared with those in cluster T3. Undernutrition is characterized by a reduction of the fat-free mass (FFM, mainly SMM) and fat mass (FM) [43]. Therefore, compared with cluster T2 and T3, cluster T1 was defined as “patients with undernutrition”.

Cluster T1 presented worse airway obstruction (FEV_1_% predicted: 56.0 (44.5, 68.0) %; Pre-FEV_1_/FVC: 55.91 (47.18, 62.97) %) than those patients in Clusters T2 and T3 (*p* < 0.05). Further, patients in cluster T1 had higher bronchodilator reversibility (BDR) (ΔFEV1: 16.15 (9.10, 30.80) %, *p* < 0.05) (Table 1).

The patients in this cluster had fewer blood eosinophils (0.19 (0.11, 0.34) × 10^9^/L, *p* < 0.05) compared with those in cluster T3. Cluster T1 had lower IgE (75.65 (33.6, 205.0) IU/mL) and sputum macrophages (34.88 (11.50, 61.25) %), but more sputum neutrophils (56.5 (31.00, 81.62) %) than those in clusters T2 and T3 (*p* < 0.05) (Table 2). Almost half of the patients (*n* = 88, 55.3%) presented with uncontrolled asthma (*p* < 0.017) (Table 1).

#### 3.2.2. Cluster T2 (Cluster 2 in the Training Set): Intermediate Level of Nutrition with Psychological Dysfunction

This cluster comprised 102 patients (18.9%) who were mostly female (*n* = 68, 66.7%). Compared with clusters T1 and cluster T3, patients in cluster T2 had an intermediate level of SMM (Figure 2). Patients in cluster T2 had higher depression (HADS-D: 6.0 (5.0, 9.0), *p* < 0.05) and anxiety scores (HADS-A: 6.0 (5.0, 8.0), *p* < 0.05) than Cluster T1 and T3 (Table 1). Cluster T2 also presented a higher prevalence of depression (HADS-D ≥ 8; *n* = 37 (36.3%), *p* < 0.001), anxiety (HADS-A ≥ 8; *n* = 35 (34.3%), *p* < 0.001), and depression and anxiety (both HADS-D and HADS-D ≥ 8; *n* = 18 (17.6%), *p* < 0.001) compared with clusters T1 and T3. About half of the patients in clusters T2 (*n* = 54, 52.9%) presented as uncontrolled asthma. These things considered, these patients had a poorer quality of life (AQLQ scores: 5.40 (5.00, 6.16), *p* < 0.05) than those in clusters T1 and T3 (Table 1).

#### 3.2.3. Cluster T3 (Cluster 3 in the Training Set): Patients with Good Nutrition

Cluster T3 included 280 (51.8%) patients. This cluster not only had a significantly higher level of BMI (23.15 (20.95, 25.33) kg/m^2^), FM (17.84 (6.27) kg, *p* < 0.05), and PBF (29.15 (6.98) %, *p* < 0.05), but also had a higher mean SMM (23.29 (4.99) kg, *p* < 0.05) and proportion of patients with higher level of SMM (76.1%, *p* < 0.001) compared with clusters T1 and T2 (Figure 2).

Cluster T3 was characterized by less airway obstruction (FEV_1_% predicted: 84.0 (72.0, 94.0) %; Pre-FEV_1_/FVC: 72.98 (65.97, 80.53) %) than those patients in clusters T1 and T2 (*p* < 0.05). Further, a lower BDR (ΔFEV_1_: 10.10 (4.58, 16.13) %, *p* < 0.05) was identified in cluster T3. Cluster T3 presented a higher prevalence of rhinitis (*n* = 175, 62.5%, *p* < 0.005) compared with the other two clusters (Table 1).

In addition, patients in cluster T3 had elevated blood eosinophils (0.27 (0.15, 0.42) × 109/L, *p* < 0.05) and IgE (164.50 (65.20, 359.00) IU/mL, *p* < 0.05) (Table 2). More than half of the patients (*n* = 174, 62.1%) presented with controlled asthma (ACQ < 0.75). Furthermore, patients in Cluster T3 had a higher (the highest) asthma quality of life questionnaire score (AQLQ; 6.25 (5.50, 6.61)) than clusters T1 and T2 (*p* < 0.05).

### 3.3. Asthma Exacerbations in the Following Year

A prospective one-year study was conducted to follow these patients in the training cohort, and a total of 479 patients (88.5%) who completed the one-year follow-up in a real-world setting were analyzed (Table 3). Compared with cluster T3, patients in cluster T2 had a higher proportion of experiencing severe exacerbation (cluster T3: 8.8% vs. cluster T2 19.0%, *p* < 0.017), hospitalization (cluster T3: 4.8% vs. cluster T2: 12.7%, *p* < 0.017), and emergency visit (cluster T3:1.6% vs. cluster T2: 7.6%, *p* < 0.017). Patients in cluster T2 also experienced a higher frequency of severe exacerbations (cluster T3: 1.38 (0.88) vs. cluster T2: 2.37 (1.64), *p* < 0.05), systemic corticosteroid bursts (cluster T3: 1.38 (1.02) vs. cluster T2: 2.17 [1.4], *p* < 0.05), hospitalizations (cluster T3: 1.08 (0.51) vs. cluster T2: 1.92 (0.79), *p* < 0.05) and emergency visits (cluster T3: 1 (0.1) vs. cluster T2: 2.5 (1.93), *p* < 0.05).

**Figure 3:**
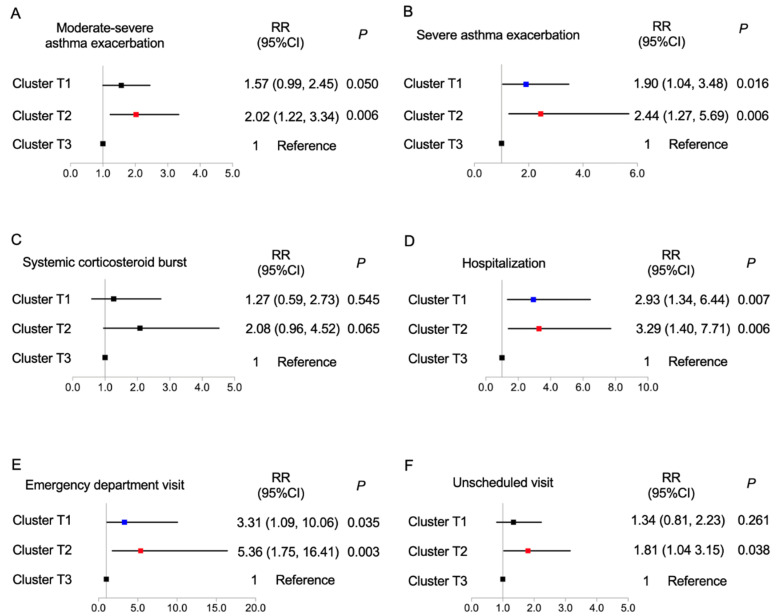
Correlations of 3 identified clusters with (**A**) moderate-to-severe exacerbation, (**B**) severe exacerbation, (**C**) systemic corticosteroid burst, (**D**) hospitalization, (**E**) emergency department visit, and (**F**) unscheduled visit; logistic regression analysis, with cluster 3 as the reference. CI, confidence interval; RR relative ratio. Blue, cluster T1; Red, cluster T2; Black, cluster T3.

We further established logistic regression models to analyze the future risk of asthma exacerbation across the pre-specified clusters (Figure 3). These analyses indicated that cluster T3 had a decreased risk of asthma exacerbation in the following year. When cluster T3 was taken as the reference, cluster T2 had a significantly increased risk of moderate-to-severe exacerbation (relative risk (RR) (95% confidence interval (CI)), 2.021 (1.22, 3.337)), severe exacerbations (2.443 (1.273, 5.685)), hospitalization (3.285 (1.400, 7.705)), emergency visit (5.361 (1.752, 16.405)), and unscheduled visit (1.805 (1.035, 3.150)). Furthermore, cluster T1 had a higher risk of severe exacerbation (RR (95% CI), 1.902 (1.040, 3.478)), hospitalization (2.937 (1.339, 6.442)), and emergency visit (3.310 (1.089, 10.057)) than cluster T3.

### 3.4. Factors Associated with Current Asthma Control and Further Exacerbation

We further explored the factors associated with current asthma control and further exacerbation in the following year (Tables S3 and S4).

### 3.5. Internal and External Validation

#### 3.5.1. Discriminant Analysis

**Figure 4:**
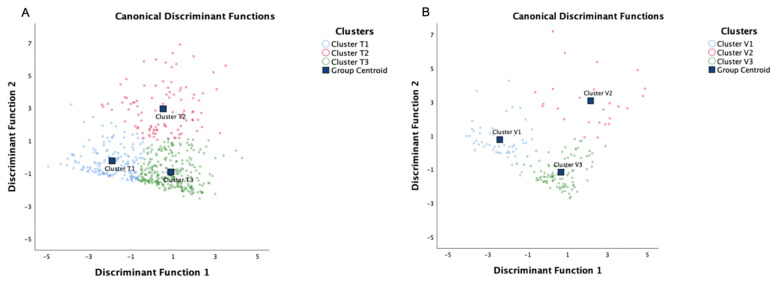
Canonical discriminant function analysis of the patient in (**A**) the training set and (**B**) the validation set. By using a stepwise method of discriminant analysis, three clusters were separated in the two sets. Cluster T, clusters identified in the training set; Cluster V, clusters identified in the validation set.

By using a stepwise method of discriminant analysis, 6 of 10 variables (age, pre-FEV_1_%, SMM, VFA, HADS-D, and HADS-A) were found to be statistically significant discriminants (Table 4). In addition, by applying the Fisher discriminant method, 2 canonical discriminant functions were generated to form a scatter plot. The clusters were well separated from each other, as shown in Figure 4A. Finally, 97.8% of patients in training set were correctly classified (Table S5).

#### 3.5.2. Cluster Analysis in Validation Set

The validation set consisted of 179 patients. We compared the clinical characteristics and body compositions (Table S6) and no significant difference was identified. Using the same algorithm to perform PCA and cluster analysis in the independent validation set also resulted in four components and three significantly different clusters, respectively (Table S7). The Kaiser–Meyer–Olkin (0.602) and the Bartlett´s Test of Sphericity (*p* < 0.001) confirmed that the cluster analysis in the validation set was appropriate. In the validation set, the PCA also identified four components: component 1 encompassed the variables BMI, FM, PBF, and VFA; component 2 encompassed sex and SMM; component 3 encompassed HADS-D and HADS-A; and component 4 encompassed age and pre-FEV_1_% (Tables S5 and S6). Figure 4 shows similar positions of the clusters between the training and validation set.

## 4. Discussion

To the best of our knowledge, this is the first study to explore asthma phenotypes by cluster analysis of BC parameters, which indicates that nutritional status evaluated by BCA can identify asthma phenotypes. As a result, we identified three asthma phenotypes in a real-world setting: “patients with undernutrition”, “intermediate level of nutrition with psychological dysfunction”, and “patients with good nutrition”. Further, our study described the clinical characteristics associated with these phenotypes, and validated the identified phenotypes associated with disease progression. Of these three phenotypes, the “patients with good nutrition” phenotype was significantly associated with a decreased risk of moderate-to-severe and severe AE compared with the other two clusters. Our study identified “BC-specific” phenotypes and highlights the importance of evaluating nutritional status in the multidimensional assessment and management of asthma, which would be of great relevance to clinical practice.

Clinical practice’s use of BMI for defining nutritional status in asthma may be limited [11,15,44,45]. To overcome the shortcomings of the BMI and to gain more information on phenotypes of asthma, BCA was necessary [46,47,48,49,50,51]. In our study, phenotypes identified by BCA had different airway and systemic inflammatory profiles. Cluster T3 comprised patients with higher blood eosinophil counts, more patients with eosinophilic asthma, and had a better prognosis. Higher peripheral blood and airway eosinophil counts suggested that these patients may have a favorable response to corticosteroids [52], hence this information is useful in guiding asthma management. Our study also provided evidence that the BMI-based categories cannot be treated as entities. Although our discriminant analysis showed that both SMM and VFA (discriminating fat-free mass and body fat distribution, respectively) were statistically significant discriminants (Table 4), the influences of SMM and VFA were different (Tables S3 and S4). Thus, BCA subdivided the crude BMI-based phenotype into more sharply divided phenotypes for asthma. The BC-specific asthma phenotypes identified in our study suggested that subtyping the asthma phenotype by using BCA may improve research, clinical practice, and potential treatment.

Previous cluster analyses using BMI to define obesity have established that the obese–asthma phenotype represents a unique set of observable characteristics [18,19,53,54]. However, these studies did not consider other important variables related to nutritional status, such as FM and SMM. Our study highlights the value of collecting more detailed additional information on body composition. In routine clinical practice, nutritional status is inadequately evaluated in asthmatics, especially in those at high risk of AE. This study identified the relationships between nutritional status evaluated by BCA with asthma control and AE. In our study, compared with the patients with undernutrition in cluster T1, those in cluster T3 were identified as having a significantly better nutritional status (higher levels of BMI, FM, PBF, and VFA) and a lower risk of uncontrolled asthma and AE in the following year. Compared with cluster T1 and T2, the proportion of patients with higher level of SMM in the cluster T3 increased significantly, which can be considered as a possible explanation for the decreased risk of uncontrolled asthma and asthma exacerbation in patients in cluster T3. That is, these findings suggested that an increased level of SMM as fat-free mass can improve the prognosis for asthma. We further explored the factors associated with current asthma control and further AE in our sample. Interestingly, significant associations of higher SMM with a decreased risk of uncontrolled asthma as well as moderate-to-severe and severe AE were identified. These findings may be explained by a previous study showing that skeletal muscle produces and releases myokines exerting metabolic and anti-inflammatory effects on the muscle itself, adipose tissue, cells of the immune system, and pancreatic islets with positive effects on insulin-induced glucose disposal [49]. These findings may suggest that the better prognosis in cluster T3 patients is due to their high level of SMM.

In addition to SMM, psychological characteristics were an important differentiating factor between clusters identified by discriminant analysis in our study. As shown in Table 1, HADS (-D and -A) values and the prevalence of depression and anxiety varied widely between the clusters: the patients in cluster T2 had more patients with depression and anxiety than the other two clusters. The importance of this finding regarding the psychological characteristic as a differentiating factor for identifying clusters in our study was similar to another cluster analysis in moderate-to-severe asthmatic populations [17]. Cluster T2 showed a significantly increased risk of moderate-to-severe and severe AE, and higher HADS-D values were associated with an increased risk of uncontrolled asthma and AE. Depression and anxiety assessed by HADS have been identified as important extra-pulmonary treatable traits of asthma [55]. Our study further indicates the importance of placing more emphasis on the assessment of psychiatric characteristics when assessing severity, therapy, and prognosis.

The main strength of our study was the use of an independent validation set to confirm the results from the training set. In addition, we quantified the phenotypes using objective measures of BC in our cluster analysis. We also recognized that this study had limitations. Firstly, our study has showed that BC-based phenotypes can lead to better or worse future AEs. However, we have only demonstrated an association—not a causation. It is possible that higher body fat causes more severe asthma (e.g., clusters with higher PBF (T1 and T2) compared to T3 have more SAE, etc.,). However, it may also be true that more severe AEs may lead to more corticosteroid treatment leading to a higher PBF. Our current analysis failed to determine the direction of the association. Therefore, we acknowledged the potential bidirectionality of the association. Secondly, only Chinese patients were enrolled, so the patients evaluated may have different BC distribution than other populations. Thirdly, although the validation of the clusters identified in this study was performed in an independent cohort, further validation in prospective cohorts including a wider variety of ethnicities is needed. Fourthly, the sample size was relatively small; multicenter and large sample studies are needed. We did not explore the distribution of those lost to follow up because of missing data resulting in a selection bias. Finally, this study did not explore the mechanism involved and further studies are required to elucidate the molecular and inflammatory mechanisms based on our study findings.

## 5. Conclusions

In conclusion, our study is the first to apply cluster analysis to identify BC-specific asthma phenotypes. We defined three distinct nutritional status-related phenotypes with distinct clinical characteristics and asthma outcomes. Our data reinforced the importance of evaluating nutritional status rather than simple BMI measurement in clinical practice to recognize asthma phenotypes and further individualize treatments with the goal of improving clinical outcomes. Evaluating nutritional status may further progress phenotype-guided management approaches. Further studies are necessary to assess the usefulness of suggesting BC-phenotypic interventions for the prevention of uncontrolled asthma and recurrent AEs.

## Figures and Tables

**Table 1:** Demographic and clinical characteristics of the included participants with asthma grouped by cluster analysis in the training set.

Variables	Cluster T1	Cluster T2	Cluster T3	Total	*F/* *χ^2^/H*	*p*-Value
*n* (%)	159 (29.4)	102 (18.9)	280 (51.8)	541	-	-
Anthropometric/asthma data						
Age, years, median (Q1, Q3)	59.0 (51.0, 68.0)	48.0 (40.0, 58.0) *	46.0 (36.0, 53.0) *	49.0 (39.0, 58.0)	67.620	<0.001
Female, *n* (%)	89 (56.0)	68 (66.7)	193 (68.9) †	350 (64.7)	7.664	0.022
BMI, kg/m^2^						
Median (Q1, Q3)	22.36 (19.97, 24.15)	22.83 (20.59, 25.01)	23.15 (20.95, 25.33) *	22.73 (20.69, 24.77)	8.196	0.017
<25, *n* (%)	133 (83.6)	76 (74.5)	205 (73.2)	414 (76.5)	7.675	0.104
25 ≤ BMI < 30, *n* (%)	23 (14.5)	21 (20.6)	58 (20.7)	102 (18.9)		
≥30, *n* (%)	3 (1.9)	5 (4.9)	17 (6.1)	25 (4.6)		
WHR, median (Q1, Q3)	0.89 (0.83, 0.93)	0.87 (0.82, 0.92)	0.87 (0.82, 0.92)	0.87 (0.82, 0.92)	4.251	0.119
Atopy, *n* (%)	52 (32.7)	32 (31.4)	155(55.4) †††	239 (44.2)	29.459	<0.001
Asthma duration, years, median (Q1, Q3)	7.0 (3.0, 16.0)	8.0 (4.0, 15.0)	6.0 (3.0 13.0)	6.0 (3.0, 15.0)	1.254	0.534
Early-onset asthma, *n* (%)	22 (13.8)	17 (16.7)	57 (20.4)	96 (17.7)	3.054	0.217
History of family asthma, *n* (%)	72 (45.3)	30 (29.4) †	89 (31.8) †	191 (35.3)	12.084	0.017
Eosinophilic asthma, *n* (%)	86 (54.1)	70 (68.6)	197 (70.4) ††	353 (65.2)	12.471	0.002
Medications						
ICS (BDP equivalent) dose, μg/day, median (Q1, Q3)	400.0 (400.0, 1000.0)	400.0 (400.0, 1000.0)	400.0 (400.0, 1000.0)	400.0 (400.0, 1000.0)	2.404	0.301
ICS/LABA, *n* (%)	91 (57.2)	58 (56.9)	161 (57.5)	310 (57.3)	0.013	0.994
Theophylline, *n* (%)	28 (17.6)	18 (17.6)	35 (12.5)	81 (15.0)	2.787	0.248
Leukotriene, *n* (%)	48 (30.2)	39 (38.2)	103 (36.8)	190 (35.1)	2.472	0.291
OCS, *n* (%)	6 (3.8)	2 (2.0)	9 (3.2)	17 (3.1)	0.739	0.691
Asthma control						
Uncontrolled asthma (ACQ scores ≥ 0.75)	88 (55.3)	54 (52.9)	106 (37.9) †††§	248 (45.8)	15.046	0.001
Health status						
AQLQ scores, median (Q1, Q3)	6.16 (5.58, 6.69)	5.40 (5.00, 6.16) *	6.25 (5.50, 6.61) **	5.96 (5.35, 6.47)	13.069	0.001
HADS-D						
Median (Q1, Q3)	1.0 (0, 1.5)	6.0 (5.0, 9.0) *	0.5 (0, 2.0) **	1.0 (0.0, 3.0)	228.027	<0.001
≥8, *n* (%)	0 (0)	37 (36.3) †††	0 (0) §§§	37 (6.8)	170.936	<0.001
HADS-A						
Median (Q1, Q3)	1.0 (0, 2.0)	6.0 (5.0, 8.0) *	1.0 (0, 2.0) **	1.0 (0.0, 4.0)	218.796	<0.001
≥8, *n* (%)	0 (0)	35 (34.3) †††	0 (0.0) §§§	35 (6.5)	161.057	<0.001
Both HADS-D and HADS-A ≥ 8, *n* (%)	0 (0)	18 (17.6) †††	0 (0.0) §§§	18 (3.3)	80.137	<0.001
SAEs in the past 12 months, *n* (%)	55 (34.6)	35 (34.3)	68 (24.3)	158 (29.2)	6.796	0.033
Spirometry						
Pre-FEV_1_, L, median (Q1, Q3)	1.43 (1.15, 1.71)	2.01 (1.62, 2.43) *	2.49 (2.12, 2.95) *^,^**	2.09 (1.56, 2.65)	225.536	<0.001
Pre-FEV_1_ % predicted, median (Q1, Q3)	56.0 (44.5, 68.0)	72.0 (58.0, 86.0) *	84.0 (72.0, 94.0) *^,^**	74.0 (59.0, 88.0)	182.736	<0.001
Pre-FEV_1_/FVC, %, median (Q1, Q3)	55.91 (47.18, 62.97)	66.02 (56.99, 75.07) *	72.98 (65.97, 80.53) *^,^**	67.19 (57.49, 76.03)	161.758	<0.001
ΔFEV_1_, %, median (Q1, Q3)	16.15 (9.10, 30.80) **	11.38 (6.30, 19.46)	10.10 (4.58, 16.13) *	11.90 (5.95, 19.39)	39.457	<0.001
ΔFEV_1_/FVC, %, median (Q1, Q3)	6.79 (1.23, 13.60)	6.90 (1.00, 12.00)	6.58 (3.57, 10.49)	6.65 (2.52, 11.61)	1.236	0.539
FeNO, ppb, median (Q1, Q3)	26.00 (16.00, 46.41)	37.50 (22.00, 71.00) *	52.50 (25.00, 96.50) *^,^ **	40.0 (21.0, 75.00)	53.732	<0.001
Comorbidities, *n* (%)						
Rhinitis	69 (43.4)	63 (61.8) ††	175 (62.5) †††	307 (56.7)	16.368	<0.001
Nasal polyps	15 (9.4)	15 (14.7)	20 (7.1)	50 (9.2)	5.108	0.078
Bronchiectasis	11 (6.9)	8 (7.8)	6 (2.1) †,§§	25 (4.6)	8.500	0.014
Sleep apnea	2 (1.3)	0 (0.0)	4 (1.4)	6 (1.1)	2.545	0.280
GERD	9 (5.7)	6 (5.9)	11 (3.9)	26 (4.8)	0.984	0.612
Diabetes	8 (5.0)	2 (2.0)	3 (1.1) †	13 (2.4)	6.331	0.042
Eczema	20 (12.6)	22 (21.6)	49 (17.5)	91 (16.8)	3.781	0.151
Body composition, mean (SD)						
FM, kg	15.24 (5.41)	16.52 (6.25)	17.84 (6.27) #	16.83 (6.13)	9.591	<0.001
PBF, %	27.24 (7.50)	27.97 (8.19)	29.15 (6.98) #	28.36 (7.41)	3.573	0.029
VFA, cm^2^	70.66 (29.84)	75.50 (34.13)	78.52 (31.63) #	75.64 (31.73)	3.139	0.044
SMM, kg	21.57 (4.07)	22.75 (4.71)	23.29 (4.99) #,##	22.69 (4.73)	6.868	0.001

Abbreviations: BMI, body mass index; WHR, waist-to-hip ratio; ICS, inhaled corticosteroid; BDP, Beclomethasone dipropionate; LABA, long-acting beta-agonist; OCS, oral corticosteroid; ACQ, asthma control questionnaire; AQLQ, asthma quality of life questionnaire; HADS-D, Hospital Anxiety and Depression scale-depression; HADS-A, Hospital Anxiety and Depression scale-anxiety; FEV_1_, forced expiratory volume in 1 s; FVC, forced vital capacity; FeNO, fractional exhaled nitric oxide; GERD, gastroesophageal reflux disease; FM, fat mass; PBF, percentage body fat; VFA, visceral fat area; SMM, skeletal muscle mass; SD, standard deviation; Q1, first quartile; Q3, third quartile. Pack years: the number of cigarettes smoked per day × years of smoking. Eosinophilic asthma: sputum eosinophil level ≥ 3% or blood eosinophil level ≥ 300 cells/mL. Uncontrolled asthma was defined as ACQ score ≥ 0.75. Kruskal–Willis Test: * *p* < 0.05 vs. cluster 1; ** *p* < 0.05 vs. cluster 2. The significance level is 0.05. Significance values have been adjusted by the Bonferroni correction for multiple tests. ANOVA Test: # *p* < 0.05 vs. cluster 1; ## *p* < 0.05 vs. cluster 2. The significance level is 0.05. Significance values have been adjusted by the LSD for multiple tests. Chi-Square Test: † *p* < 0.017; †† *p* < 0.005; ††† *p* < 0.001 vs. cluster 1, with the Bonferroni correction; § *p* < 0.017; §§ *p* < 0.005; §§§ *p* < 0.001 vs. cluster 2, with the Bonferroni correction.

**Table 2:** Inflammatory characteristics of the included patients with asthma grouped by cluster analysis in the training set.

Variables	Cluster T1	Cluster T2	Cluster T3	Total	*H*	*p*-Value
*n* (%)	159 (29.4)	102 (18.9)	280 (51.8)	541	-	-
Peripheral blood, median (Q1, Q3)						
Eosinophils, × 10^9^/L	0.19 (0.11, 0.34)	0.24 (0.13, 0.38)	0.27 (0.15, 0.42) *	0.21 (0.12, 0.33)	11.581	0.003
Neutrophils, × 10^9^/L	3.37 (2.57, 4.36)	3.02 (2.46, 4.21)	3.32 (2.67, 4.13)	3.27 (2.63, 3.92)	0.854	0.653
Lymphocytes, × 10^9^/L	1.68 (1.39, 2.06)	1.58 (1.31, 1.88)	1.67 (1.40, 1.92)	1.71 (1.40, 2.07)	4.094	0.129
Monocytes, × 10^9^/L	0.36 (0.27, 0.48)	0.31 (0.24, 0.42)	0.33 (0.27, 0.41)	0.33 (0.27, 0.42)	4.977	0.083
Basophils, × 10^9^/L, median (Q1, Q3)	0.03 (0.02, 0.05)	0.04 (0.02, 0.05)	0.04 (0.02, 0.05)	0.03 (0.02, 0.05)	0.168	0.920
IgE, IU/mL	75.65 (33.60, 205.00)	126.00 (41.97, 304.95) *	164.50 (65.20, 359.00) *	92.85 (36.67, 302.45)	18.264	<0.001
Sputum, median (Q1, Q3)						
Eosinophils, %	0.25 (0, 1.75)	0.25 (0, 1.50)	0.50 (0.25, 1.50)	0.25 (0, 3.00)	2.212	0.331
Neutrophils, %	56.5 (31.00, 81.62)	35.00 (13.25, 68.00) *	32.88 (15.13, 62.25) *	42.63 (17.00, 71.88)	14.869	0.001
Lymphocytes, %	0.50 (0, 1.00)	0.50 (0.25, 1.50)	0.50 (0.25, 1.50)	0.50 (0.25, 1.25)	4.272	0.118
Macrophages, %	34.88 (11.50, 61.25)	47.88 (17.00, 81.50) *	58.13 (28.63, 78.99) *	46.25 (19.38, 73.88)	15.745	<0.001

Abbreviations: Ig, immunoglobulin; Q1, first quartile; Q3, third quartile. Kruskal–Willis Test: * *p* < 0.05 vs. cluster 1. The significance level is 0.05. Significance values have been adjusted by the Bonferroni correction for multiple tests.

**Table 3:** Asthma exacerbation within the 12-month follow-up period in the training set.

Outcomes	Cluster T1	Cluster T2	Cluster T3	Total	*χ^2^*/*H*	*p*-Value
*n* (%)	149 (31.1)	79 (16.5)	251 (52.4)	479		
Moderate-to-severe asthma exacerbation						
*n* (%)	39 (26.2)	24 (30.4)	61 (24.3)	124 (25.9)	1.166	0.558
Mean (SD)	2.53 (3.01)	2.39 (1.84)	2.03 (1.64)	2.29 (2.23)	1.467	0.480
Severe asthma exacerbation						
*n* (%)	23 (15.4)	15 (19.0)	22 (8.8) §	60 (12.6)	7.230	0.027
Mean (SD)	1.92 (1.67)	2.37 (1.64)	1.38 (0.88) **	1.85 (1.46)	9.178	0.010
Systemic corticosteroid burst						
*n* (%)	13 (8.7)	10 (12.7)	16 (6.4)	39 (8.1)	3.271	0.195
Mean (SD)	1.58 (0.79)	2.17 (1.4)	1.38 (1.02) **	1.68 (1.12)	6.025	0.049
Hospitalization						
*n* (%)	16 (10.7)	10 (12.7)	12 (4.8) §	38 (7.9)	7.435	0.024
Mean (SD)	1.24 (0.56)	1.92 (0.79) *	1.08 (0.51) **	1.39 (0.7)	9.981	0.007
Emergency department visit						
*n* (%)	9 (6.0)	6 (7.6)	4 (1.6) †,§	19 (4.0)	8.123	0.017
Mean (SD)	2.11 (2.62)	2.5 (1.93)	1 (0.1) **	2 (2.05)	6.461	0.040
Unscheduled visit						
*n* (%)	24 (16.1)	15 (19.0)	46 (18.3)	85 (17.7)	0.415	0.812
Mean (SD)	2.43 (2.26)	2.15 (1.53)	2.02 (1.58)	2.18 (1.8)	0.780	0.677

Chi-Square Test: † *p* < 0.017 vs. cluster 1, with the Bonferroni correction; § *p* < 0.017 vs. cluster 2, with the Bonferroni correction. Kruskal–Willis Test: * *p* < 0.05 vs. cluster 1; ** *p* < 0.05 vs. cluster 2. The significance level is 0.05. Significance values have been adjusted by the Bonferroni correction for multiple tests.

**Table 4:** Canonical discriminant function analysis in the training set.

Step	Variables	Tolerance	Sig. of F to Remove	Wilks’ Lambda
1	HADS-D	1.000	<0.001	
2	HADS-D	0.993	<0.001	0.659
	Pre-FEV_1_%	0.993	<0.001	0.423
3	HADS-D	0.991	<0.001	0.498
	Pre-FEV_1_%	0.984	<0.001	0.302
	Age	0.991	<0.001	0.277
4	HADS-D	0.880	<0.001	0.222
	Pre-FEV_1_%	0.976	<0.001	0.240
	Age	0.989	<0.001	0.222
	HADS-A	0.883	<0.001	0.209
5	HADS-D	0.880	<0.001	0.204
	Pre-FEV_1_%	0.960	<0.001	0.226
	Age	0.907	<0.001	0.217
	HADS-A	0.877	<0.001	0.194
	VFA	0.902	<0.001	0.168
6	HADS-D	0.878	<0.001	0.200
	Pre-FEV_1_%	0.957	<0.001	0.222
	Age	0.906	<0.001	0.212
	HADS-A	0.876	<0.001	0.190
	VFA	0.895	<0.001	0.163
	SMM	0.984	<0.001	0.155

Abbreviations: FEV_1_, forced expiratory volume in 1 s; HADS-A, Hospital Anxiety and Depression scale-anxiety; HADS-D, Hospital Anxiety and Depression scale-depression; VFA, visceral fat area; SMM, skeletal muscle mass.

## Supplementary Materials

The following supporting information can be downloaded at: https://www.mdpi.com/article/10.3390/nu14122525/s1, Table S1. Rotated component matrix for training set; Table S2. Pseudo-F Statistic and Pseudo-T2 Statistic for the different number of clusters in our study; Table S3. Risk factors associated with exacerbation, hospitalization, emergency department visit, systemic corti-costeroid burst and unscheduled visit; Table S4. Factors associated with uncontrolled asthma (ACQ ≥ 0.75) in the training set; Table S5. Classification results in discriminant analysis in the training set; Figure S1. Average silhouette width (A) and silhouette plot (B) in the training set Table S6. Demo-graphic and clinical character-istics of the included participants with asthma in the training and validation set; Table S7. Rotated component matrix for validation set. References [56,57,58,59,60,61,62,63,64,65,66,67,68,69,70] are cited in the Supplementary Materials.
