# Supplementary material for: Body Composition-Specific Asthma Phenotypes: Clinical Implications
Source: Nutrients. 2022 Jun 17;14(12):2525. doi: 10.3390/nu14122525 (PMC9229860; doi:10.3390/nu14122525)
Supplement: Supplementary file 1 [file nutrients-14-02525-s001.zip › nutrients-1730286-Supplementary.pdf]

Supplementary Materials

These supplementary materials have been included by the authors to provide readers with additional information about the methods and results. The components of the supplementary materials are as follows:

Body composition-specific asthma phenotypes: clinical implications

1. Methods

2. Results

2.1. Table S1. Rotated component matrix for training set

2.2. Table S2. Pseudo-F Statistic and Pseudo-T2 Statistic for the different number of clusters in our study

2.3. Table S3. Risk factors associated with exacerbation, hospitalization, emergency department visit, systemic corticosteroid burst and unscheduled visit

2.4 Table S4. Factors associated with uncontrolled asthma (ACQ≥0.75) in the training set

2.5 Table S6. Demographic and clinical characteristics of the included participants with asthma in the training and validation set

2.6 Table S7. Rotated component matrix for validation set

2.7 Table S5. Classification results in discriminant analysis in the training set

2.8 Figure S1. Average silhouette width (A) and silhouette plot (B) in the training set

References

1. METHODS

1.1 Multidimensional assessment and data collection

Data on demographics and clinical characteristics were collected using standardized case report forms. Further assessments included:

Early-onset asthma, asthma control and asthma-related health-status

Early-onset asthma was defined as an asthma diagnosis before the age of 12 years [1]. The Asthma Control Questionnaire-6 (ACQ-6) [2,3] was used for assessing asthma control. Scores range between 0 and 6, where ACQ <0.75 and $\geqslant 0.75$ are indicative of controlled and partially/uncontrolled asthma, respectively [4]. Health-related quality of life was assessed using the Asthma Quality of Life Questionnaire (AQLQ). Scores range between 0 and 7, with higher scores indicating better the asthma-related health-status [5]. Psychological dysfunction due to anxiety or depression was assessed by the 14-item Hospital Anxiety and Depression Scale (HADS) [6,7].

Asthma exacerbations

Severe asthma exacerbation (AE) [8,9] was defined as worsening of asthma symptoms that led to one of the following: ≥3 days of oral corticosteroids (OCS) treatment or a temporary increase in their OCS maintenance dosage; or an emergency department (ED) or intensive care unit (ICU) visit requiring OCS; or an asthma-specific hospitalization. Moderate asthma exacerbation [8,9] was defined as any increase in rescue bronchodilator use for at least 2 days or any temporary increase in inhaled corticosteroids (ICS), or an emergency department visit or an unscheduled visit while

not requiring OCS due to asthma symptoms worsening. AEs during the previous year were recorded.

Anthropometrics

Anthropometrics [10,11] (weight, height, body mass index [BMI]) and body composition (BC) of the participants were measured by trained researchers. Weight was recorded to the nearest 0.1 kg using a digital scale and height to the nearest 0.1 cm, using a telescopic height measuring instrument. Waist circumference (WC) and hip circumference (HC) were measured using an inelastic tape, precision 0.1 cm, range 1–150 cm at the navel and at the maximum posterior protuberance of the buttocks, respectively, with the patient in a standing position [12,13] Only light indoor clothing was worn.

Spirometry and fractional exhaled nitric oxide

Spirometry (model 6200; SensorMedics Corp., Yorba Linda, CA) was performed according to the American Thoracic Society and European Respiratory Society (ATS/ERS) recommendations [14]. Forced expiratory volume in one second (FEV_1) and forced vital capacity (FVC) were measured before and 15 minutes after 400 µg salbutamol delivered by metered-dose inhaler and spacer. Fractional exhaled nitric oxide (FeNO) was measured by the NIOX analyzer (Aerocrine, Solna, Sweden) according to guidelines from the ATS [15].

Atopy and skin prick tests

Atopy was confirmed by a positive response to one or more allergen using a skin-prick testing (SPT) with common aeroallergens as described in a recently published study and GINA [16,17].

Sputum induction and peripheral blood collection and detection

Sputum induction and processing and peripheral blood collection were performed based on standard methods as described in our previous studies [16,18].

Sputum induction and processing

Sputum induction and processing were performed based on standard methods as described in our previous studies [16,18]. In brief, sputum was induced after the pre-treatment of 400 mcg salbutamol (GSK) using 4.5% saline atomized by an ultrasonic nebulizer (Cumulus; HEYER Medical AG, Bad Ems, Germany). If FEV_1 was ≤ 40% of predicted, sputum was induced with 0.9% saline after it was deemed safe by the supervising physician. Sputum plugs were collected and an aliquot of 200 μL of sputum plug was quick-frozen immediately by liquid nitrogen and stored at −80°C for subsequent metabolism analysis. Further, a volume of 1% dithiothreitol (SPUTOLYSIN Reagent; Calbiochem®, San Diego, CA, USA) was added equal to 4 times the remaining sputum at 1,500 rpm for 10 minutes, and the sputum supernatant was aspirated and stored immediately at −80°C for subsequent detection. Total and differential cell counts were performed with centrifugation-smear (CYTOPRO 7620; WESCOR®, Inc., South Logan, UT, USA) and staining preparation by well-trained

Chinese and Australia lab researchers.

Peripheral blood collection and detection

Fasting intravenous blood samples were collected for blood cell counts (Sysmex XN-9000 hematology analyzer; Sysmex Corporation, Kobe, Japan) using standard morphological criteria and analysis of total serum IgE levels (Beckman Immage 800 immunology analyzer; Beckman Coulter Inc., Brea, Calif), and 5.0 IU/mL was the minimum detectable level.

1.2 Statistical analysis

Continuous variables are presented as mean (standard deviation) or median (interquartile range) according to the data distribution. Categorical variables are summarized as frequencies and proportions. Based on the multidimensional assessment of asthma, a total of 366 variables including measurements of BC were collected and recorded. The variables were then imputed by using the multiple imputation method (multilevel and generalized linear regression) [19,20]. Variable selection process was performed as mentioned in our previous study [19]. Briefly, redundant (n = 167) or irrelevant (n = 113) variables were firstly rejected based on demographic and clinical relevance, and the purpose of this study. Secondly, twenty-five variables were selected for evaluation by factor analysis with orthogonal varimax rotation [21,22]. Thirdly, based on the pattern of loading, variables with a value > 0.5 were initially selected for each factor [21,23]. Fourthly, we created a correlation matrix of the variables using the correlation coefficient [24,25]. For initially selected variables based on loading value with a correlation coefficient > 0.6, the selected variable for principal component analysis (PCA) in the next step was based on a consideration of the clinical significance of the variables [25]. Finally, 10 variables were selected for PCA based on the pattern of loading, correlation coefficient, and clinical perspective, including sex (female=1), age (years), pre-FEV_1 (%), HADS-A (scores), HADS-D (scores), BMI (kg/m^2), FM (kg), PBF (%), VFA (cm^2), and SMM (kg).

Principal component analysis (PCA)

Reducing the dimensionality of the data prior to clustering algorithms reduces the risk of overfitting. Thus, a PCA with varimax rotation was performed to merge the variables of interest into a multivariate component. PCs were constructed for the patients in training set. The appropriate number of PCs was selected by analysis of the Scree plot, with a requirement that retained PCs explain at least 70% of data variance, and that each PC have an eigenvalue > 1.0 according to the Kaiser criterion [26-29]. In the training set, the 10 variables were restructured to four PCs that captured 85.406% of the variance within the dataset (PC1 35.083%, PC2 20.349%, PC3 17.164% and PC4 12.810%). In the validation set, the 10 variables were restructured to four PCs that captured 86.547% of the variance within the dataset (PC1 35.273%, PC2 20.591%, PC3 17.719% and PC4 12.964%). The Kaiser-Meyer-Olkin (KMO) measures of sampling adequacy test and Bartlett's Test of Sphericity were used to confirm whether the PCA was appropriate for these variables [30].

Cluster analysis

Cluster analysis was conducted by applying a 2-step process using the four PCs identified in the PCA [31,32] as described in our previous studies [19,20]. Firstly, Ward's hierarchical clustering method was performed using an agglomerative (bottom-up) approach and Ward's linkage. At each generation of clusters, samples were merged into larger clusters to minimize the within-cluster sum of squares or to maximize the between-cluster sum of squares [33]. An iterative approach was then used over a range of clusters (three clusters up to a maximum of five clusters) to define the optimal

number of clusters to characterize the data. Secondly, the optimum number of clusters was determined by the maximized pseudo-F statistic [34] and the Pseudo-T2 statistic [35-37]. Thirdly, a silhouette plot was used to validate the results of the cluster analysis. Fourthly, cluster numbers were decided, a K-means cluster analysis was used to cluster cases to centroids by using the prespecified number of clusters. The stability of the clusters was tested using a repeated K-means clustering with a random sample containing 50% of the cases [30,38]. Stepwise discriminant analysis was performed to identify variables discriminating between the prespecified clusters.

Other analyses

Differences between clusters were then assessed for demographic and clinical data. For comparison between groups, normality of data was assessed using a Kolmogorov-Smirnov test or a Shapiro-Wilk test. The between-cluster differences were analyzed by the Chi-square test, ANOVA, or Kruskal-Wallis test. Multiple comparisons between groups were analyzed by Bonferroni or LSD tests, according to their normal distribution. Correlations were analyzed using Pearson or Spearman's coefficients according to their normal distribution. The association analyses were performed using multiple logistic regressions between the dependent outcomes (Uncontrolled asthma [ACQ-6$\geqslant$0.75] and AE in the following year) and the clinical and BC characteristics included in the cluster analysis: sex (female=1), age (years), pre-FEV_1 (%), HADS-A (scores) and HADS-D (scores), BMI (kg/m^2), FM (kg), PBF (%), VFA (cm^2), and SMM (kg). The stepwise forward process (Forward conditional) was used to include variables

(with P<0.20 in the univariate analysis) into the model. Statistical analyses were carried out using SPSS version 23.0 (IBM, Armonk, NY). P-value less than 0.05 was considered statistically significant. P-values may be adjusted for multiple comparisons of the clusters.

2.RESULTS

2.1 **Table S1**. Rotated component matrix for training set

Table S1. Rotated component matrix for training set*				
Variables	Component 1	Component 2	Component 3	Component 4
Age	0.158	0.045	-0.060	**-0.771**
Sex	0.168	**-0.898**	0.007	0.205
BMI	**0.863**	0.374	0.011	0.006
FM	**0.986**	0.039	0.004	0.005
PBF	**0.876**	-0.435	-0.011	-0.052
VFA	**0.962**	-0.042	0.017	-0.086
SMM	0.175	**0.940**	0.013	0.118
HADS-D	-0.001	0.015	**0.921**	0.055
HADS-A	0.017	-0.008	**0.926**	-0.026
Pre-FEV_1%	0.075	-0.010	-0.032	**0.800**
*Extraction method: Principal component analysis. Rotation Method: Varimax with Kaiser Normalization.<br>Abbreviations: BMI, body mass index; FM, fat mass, PBF, percentage body fat; VFA, visceral fat area; SMM, skeletal muscle mass, HADS-D, Hospital Anxiety and Depression scale-depression; HADS-A, Hospital Anxiety and Depression scale-anxiety; FEV_1, forced expiratory volume in 1 s.				

2.2 **Table S2**. Pseudo-F Statistic and Pseudo-T2 Statistic for the different number of clusters in our study

Table S2. Pseudo-F Statistic and Pseudo-T2 Statistic for the different number of clusters in our study		
Number of clusters	Pseudo-F statistic	Pseudo-T2 statistic
3	124	70.6

4	118	112
5	118	75.9

2.3 Table S3. Risk factors associated with exacerbation, hospitalization, emergency department visit, systemic corticosteroid burst and unscheduled visit

Table S3. Risk factors associated with exacerbation, hospitalization, emergency department visit, systemic corticosteroid burst and unscheduled visit				
Variables	Univariate analysis		Multiple logistic final model	
	RR (95% CI)	*P*-value	RR $_{adj}$ (95% CI)	*P*-value
Moderate-to-severe asthma exacerbation				
Sex, female	1.105 (0.936-1.305)	0.237		
Age, years	1.006 (1.001-1.012)	0.028#		
Pre-FEV$_1$%	0.997 (0.993-1.001)	0.100#		
HADS-D, score	1.039 (1.010-1.068)	0.007#	1.083 (1.056-1.111)	<0.001*
HADS-A, score	1.080 (1.053-1.108)	<0.001#		
BMI, kg/m^2	1.002 (0.980-1.025)	0.835		
FM, kg	1.001 (0.988-1.014)	0.858		
PBF, %	1.009 (0.998-1.020)	0.106#		
VFA, cm^2	1.002 (1.000-1.005)	0.053#	1.003 (1.001-1.006)	0.012*
SMM, kg	0.971 (0.955-0.988)	0.001#	0.967 (0.951-0.985)	<0.001*
Severe asthma exacerbation				
Sex, female	0.967 (0.777-1.202)	0.760		
Age, years	1.016 (1.009-1.023)	<0.001#	1.014 (1.007-1.022)	<0.001*

Pre-FEV_1%	0.992 (0.987-0.997)	0.001#		
HADS-D, score	1.096 (1.063-1.130)	<0.001#	1.105 (1.071-1.140)	<0.001*
HADS-A, score	1.054 (1.018-1.092)	0.003#		
BMI, kg/m^2	1.041 (1.011-1.072)	0.007#		
FM, kg	1.016 (0.999-1.033)	0.059#		
PBF, %	1.021 (1.006-1.035)	0.005#		
VFA, cm^2	1.006 (1.003-1.009)	<0.001#	1.006 (1.003-1.009)	<0.001*
SMM, kg	0.977 (0.955-1.000)	0.049#	0.974 (0.952-0.997)	0.028*
Systemic corticosteroid burst				
Sex, female	0.849 (0.650-1.109)	0.230		
Age, years	1.013 (1.004-1.022)	0.003#	1.014 (1.005-1.023)	0.002*
Pre-FEV_1%	0.994 (0.988-1.000)	0.058#		
HADS-D, score	1.080 (1.041-1.122)	<0.001#	1.085 (1.045-1.127)	<0.001*
HADS-A, score	1.064 (1.019-1.110)	0.005#		
BMI, kg/m^2	1.033 (0.996-1.071)	0.080#		
FM, kg	1.008 (0.987-1.029)	0.456		
PBF, %	1.009 (0.991-1.027)	0.339		
VFA, cm^2	1.002 (0.998-1.006)	0.261		
SMM, kg	1.004 (0.977-1.032)	0.762		
Hospitalization				
Sex, female	0.911 (0.694-1.196)	0.501		
Age, years	1.025 (1.017-	<0.001#	1.025 (1.016-	<0.001*

	1.034)		1.034)	
Pre-FEV_1%	0.991 (0.984-0.997)	0.003#		
HADS-D, score	1.091 (1.052-1.133)	<0.001#	1.105 (1.063-1.148)	<0.001*
HADS-A, score	1.072 (1.028-1.119)	0.001#		
BMI, kg/m^2	1.075 (1.038-1.114)	<0.001#		
FM, kg	1.027 (1.007-1.048)	0.009#		
PBF, %	1.028 (1.009-1.046)	0.003#		
VFA, cm^2	1.008 (1.005-1.012)	<0.001#	1.007 (1.003-1.011)	<0.001*
SMM, kg	0.988 (0.960-1.017)	0.405		
Emergency department visit				
Sex, female	0.850 (0.599-1.206)	0.363		
Age, years	1.026 (1.015-1.037)	<0.001#	1.023 (1.011-1.035)	<0.001*
Pre-FEV_1%	0.990 (0.981-0.998)	0.012#		
HADS-D, score	1.143 (1.095-1.193)	<0.001#	1.168 (1.116-1.223)	<0.001*
HADS-A, score	1.061 (1.003-1.121)	0.038#		
BMI, kg/m^2	1.045 (0.998-1.095)	0.063#		
FM, kg	1.050 (1.025-1.077)	<0.001#		
PBF, %	1.057 (1.032-1.083)	<0.001#		
VFA, cm^2	1.014 (1.009-1.019)	<0.001#	1.014 (1.009-1.019)	<0.001*
SMM, kg	0.960 (0.923-0.999)	0.042#	0.956 (0.919-0.994)	0.023*
Unscheduled visit				
Sex, female	1.412 (1.163-1.713)	<0.001#		

Age, years	1.003 (0.997-1.009)	0.305		
Pre-FEV$_1$%	1.001 (0.997-1.006)	0.587		
HADS-D, score	1.055 (1.026-1.085)	<0.001#	1.058 (1.028-1.088)	<0.001*
HADS-A, score	1.040 (1.009-1.073)	0.012#		
BMI, kg/m^2	0.972 (0.947-0.998)	0.035#		
FM, kg	0.995 (0.981-1.010)	0.532		
PBF, %	1.009 (0.997-1.021)	0.145#		
VFA, cm^2	1.001 (0.998-1.004)	0.423		
SMM, kg	0.956 (0.936-0.975)	<0.001#	0.955 (0.935-0.974)	<0.001*

Abbreviations: FEV$_1$, forced expiratory volume in 1 s; HADS-D, Hospital Anxiety and Depression scale-depression; HADS-A, Hospital Anxiety and Depression scale-anxiety; BMI, body mass index; FM, fat mass; PBF, percentage body fat; VFA, visceral fat area; SMM, skeletal muscle mass.
# $P<0.20$; *$P<0.05$.

2.4 Table S4. Factors associated with uncontrolled asthma (ACQ≥0.75) in the training set

Table S4. Factors associated with uncontrolled asthma (ACQ⩾0.75) in the training set

Variables	Univariate analysis		Multiple logistic final model	
	OR (95% CI)	*P*-value	OR $_{adj}$ (95% CI)	*P*-value
Sex, female	0.923 (0.799, 1.067)	0.280		
Age, years	1.006 (1.000, 1.011)	0.033#		
Pre-FEV$_1$%	0.969 (0.965, 0.972)	<0.001#	0.969 (0.965, 0.973)	<0.001*
HADS-D, score	1,077 (1.051, 1.103)	<0.001#		
HADS-A, score	1.133 (1.104, 1.163)	<0.001#	1.135 (1.105, 1.167)	<0.001*
BMI, kg/m^2	0.982 (0.962, 1.001)	0.068#		

FM, kg	0.997 (0.986, 1.009)	0.648		
PBF, %	1,002 (0.993, 1.011)	0.680		
VFA, cm^2	1.000 (0.998, 1.003)	0.655		
SMM, kg	0.983 (0.969, 0.998)	0.025#	0.982 (0.968, 0.997)	0.019*
Abbreviations: FEV_1, forced expiratory volume in 1 s; HADS-D, Hospital Anxiety and Depression scale-depression; HADS-A, Hospital Anxiety and Depression scale-anxiety; BMI, body mass index; FM, fat mass; PBF, percentage body fat; VFA, visceral fat area; SMM, skeletal muscle mass.<br># $P<0.20$; *$P<0.05$.				

2.5 Table S6. Demographic and clinical characteristics of the included participants with asthma in the training and validation set

Table S5. Demographic and clinical characteristics of the included participants with asthma in the training and validation set				
Variables	Training set	Validation set	$\chi^2/Z/t$	*P*-value
n (%)	541	179	-	-
Anthropometric /asthma data				
Age, years, median (Q1, Q3)	49.0 (39.0, 58.0)	49.0 (37.0, 58.0)	-0.777	0.437
Female, n (%)	350 (64.7)	122 (68.2)	0.714	0.398
BMI, kg/m^2, median (Q1, Q3)	22.73 (20.69, 24.77)	22.79 (20.95, 24.69)	-0.214	0.831
Pack years, median (Q1, Q3) ¶	19.0 (6.5, 31.0)	17.5 (6.0, 30.0)	-0.244	0.807
Atopy, n (%)	239 (44.2)	75 (41.9)	0.284	0.594
Early-onset asthma, n (%)	96 (17.7)	31 (17.4)	0.010	0.920
History of family asthma, n (%)	191 (35.3)	53 (29.6)	1.948	0.163
Asthma control				
Uncontrolled asthma (ACQ score ≥0.75)	248 (45.8)	81 (45.3)	0.019	0.891
Health status				
AQLQ scores, median (Q1, Q3)	5.96 (5.35, 6.47)	5.94 (5.19, 6.35)	-0.882	0.378
HADS-D, median (Q1, Q3)	1.0 (0.0, 3.0)	1.0 (0.0, 3.0)	-1.444	0.149

Table S5. Demographic and clinical characteristics of the included participants with asthma in the training and validation set				
Variables	Training set	Validation set	χ^2/Z/t	*P*-value
HADS-A, median (Q1, Q3)	1.0 (0.0, 4.0)	1.0 (0.0, 4.0)	-1.032	0.302
Spirometry				
Pre-FEV_1, L, median (Q1, Q3)	2.09 (1.56, 2.65)	2.11(1.53, 2.72)	-0.291	0.771
Pre-FEV_1 % predicted, median (Q1, Q3)	74.0 (59.0, 88.0)	75.0 (60.0, 89.0)	-0.373	0.709
Pre-FEV_1/FVC, %, median (Q1, Q3)	67.19 (57.49, 76.03)	68.18 (57.73, 76.25)	-0.716	0.474
FeNO, ppb, median (Q1, Q3)	40.0 (21.0, 75.0)	42.0 (20.0, 74.0)	-0.216	0.829
Body composition, mean (SD)				
FM, kg	16.83 (6.13)	17.23 (5.09)	0.648	0.421
PBF, %	28.36 (7.41)	29.29 (6.41)	2.262	0.133
VFA, cm^2	75.64 (31.73)	75.31 (30.04)	0.015	0.904
SMM, kg	22.69 (4.73)	22. 44 (4.86)	0.366	0.546

Abbreviations: BMI, body mass index; ACQ, asthma control questionnaire; AQLQ, asthma quality of life questionnaire; HAD-D, Hospital Anxiety and Depression scale-depression; HADS-A, Hospital Anxiety and Depression scale-anxiety; FEV_1, forced expiratory volume in 1 s; FVC, forced vital capacity; FeNO, fractional exhaled nitric oxide; FM, fat mass; PBF, percentage body fat; VFA, visceral fat area; SMM, skeletal muscle mass; SD, standard deviation; Q1, first quartile; Q3, third quartile.

¶Pack years: the number of cigarettes smoked per day×years of smoking.

Uncontrolled asthma was defined as ACQ score ≥0.75.

2.6 **Table S7.** Rotated component matrix for validation set

Table S6. Rotated component matrix for validation set*				
Variables	Component 1	Component 2	Component 3	Component 4
Age	0.164	0.105	0.023	**-0.773**
Sex	0.138	**-0.895**	0.023	0.260
BMI	**0.870**	0.374	-0.014	0.025
FM	**0.983**	0.078	-0.012	0.046

PBF	**0.879**	-0.423	-0.036	-0.027
VFA	**0.960**	-0.012	0.000	-0.085
SMM	0.193	**0.943**	0.000	0.118
HADS-D	0.004	0.013	**0.943**	0.058
HADS-A	-0.038	-0.030	**0.940**	-0.051
Pre-FEV_1%	0.121	0.016	0.027	**0.798**

*Extraction method: Principal component analysis. Rotation Method: Varimax with Kaiser Normalization.
Abbreviations: BMI, body mass index; FM, fat mass; PBF, percentage body fat; VFA, visceral fat area; SMM, skeletal muscle mass; HADS-D, Hospital Anxiety and Depression scale-depression; HADS-A, Hospital Anxiety and Depression scale-anxiety; FEV_1, forced expiratory volume in 1 s.

2.7 **Table S5.** Classification results in discriminant analysis in the training set

Table S7. Classification results in discriminant analysis in the training set

		Predicted clusters n (%)			Total
		Cluster T1	Cluster T2	Cluster T3	
Actual clusters n (%)	Cluster T1	155 (97.5)	1 (0.6)	3 (1.9)	159
	Cluster T2	0 (0)	102 (100.0)	0 (0)	102
	Cluster T3	7 (2.5)	1 (0.4)	272 (97.1)	280
Total		162	104	275	541 (100)

2.8 **Figure S1.** Average silhouette width (A) and silhouette plot (B) in the training set

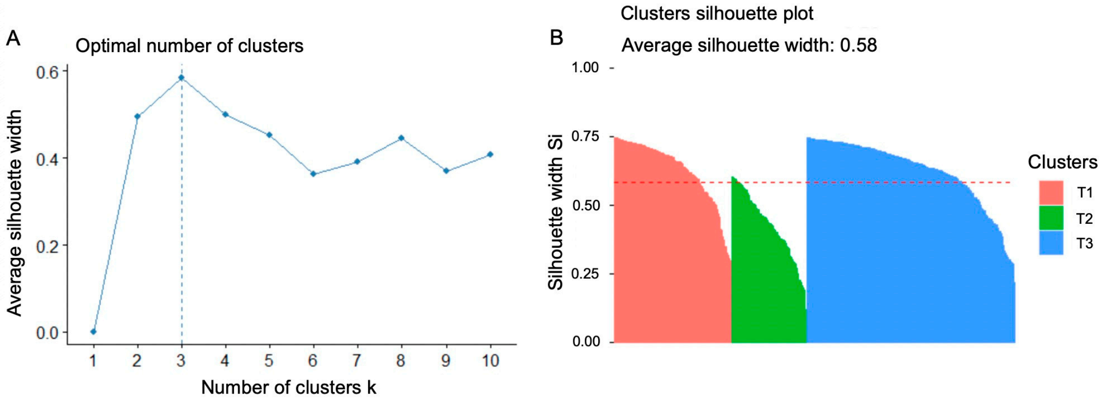

Figure S1. Average silhouette width (A) and silhouette plot (B) in the training set
